# Supplementary material for: The Systems Biology Research Tool: evolvable open-source software
Source: BMC Syst Biol. 2008 Jun 29;2:55. doi: 10.1186/1752-0509-2-55 (PMC2446383; doi:10.1186/1752-0509-2-55)
Supplement: Additional file 1 — SBRT Archive. An archive of the current version of the Systems Biology Research Tool. [file 1752-0509-2-55-S1.zip › sbrt-1.4.0/doc/developers_guide/api/sbrt/shell/io/fba/package-frame.html]

sbrt.shell.io.fba


sbrt.shell.io.fba

Interfaces 
  
*FbaOptOutputFile*

Classes 
  
CofoofSource
  
ColumnOrFbaOptOutputFile
  
FluxCapInputFile
  
FluxIntervalInputFile
  
RofoofSource
  
RowOrFbaOptOutputFile
  
RxnNamExprSetInputFile
  
SerFbaOptOutputFile
  
SfoofSource
